# Psychometric validation of the self-identification of having a mental illness (SELF-I) scale and the relationship with stigma and help-seeking among young people

**DOI:** 10.1007/s00127-018-1602-2

**Published:** 2018-10-04

**Authors:** Sara Evans-Lacko, Susanne Stolzenburg, Petra C. Gronholm, Wagner Ribeiro, Marianna York-Smith, Georg Schomerus

**Affiliations:** 1 Personal Social Services Research Unit, London School of Economics and Political Science, Houghton Street, London, WC2A 2AE UK; 2 Department of Psychiatry, Universitätsmedizin Greifswald, Ellernholzstr. 2, 17475 Greifswald, Germany

**Keywords:** Stigma, Mental health, Mental disorder, Scales, Adolescence, Youth, Lay understanding, Stereotype

## Abstract

**Purpose:**

Self-identification of having a mental illness has been shown to be an important factor underpinning help-seeking behaviour and may mediate the relationship between personal stigma and mental health service use. This study validates a new scale for the self-identification of having a mental illness among a non-clinical, community cohort of young people in the UK.

**Methods:**

Following consultation with a group of young person experts with experience of mental health problems, we evaluated the psychometric properties of the self-identification of mental illness scale (SELF-I) among 423 young people aged 13–24 years who are part of an ongoing prospective community cohort. We performed test retest reliability among a subset of 53 participants. Psychometric validation for the scale used measures of Cronbach’s alpha and Pearson’s correlation coefficient. Item performance was assessed along and in relation with each covariate.

**Results:**

The SELF-I demonstrated robust psychometric properties including high test–retest reliability (0.95) and good internal consistency (0.87 as determined by the Cronbach’s alpha). The inter-total correlations for each item, which ranged from 0.62 to 0.74, supported keeping all items in the scale. Reporting greater psychiatric symptomatology via the SDQ (*β*: 0.82 95% confidence interval 0.40, 1.23), psychotic-like experiences (*β*: 0.37 95% confidence interval 0.14, 0.59), and use of mental health services (*β*: 0.92 95% confidence interval 0.71, 1.13) were associated with a greater self-perception as having a mental illness (*p* < 0.05), providing evidence of convergent validity. As expected, we found that less intended stigmatising behaviour was associated with greater self-perceptions of having a mental illness (*B*: 0.18, 95% CI 0.07, 0.28).

**Conclusions:**

The SELF-I scale provides a method to gather insight into how young people, who may not identify as service users, perceive their own mental state and potential risk for developing a mental illness. This can be important for understanding perceived need for help and likelihood of using services among those with mental health problems.

## Introduction

Significant mental health symptoms, impairment, and disability often precede accessing support from a mental health professional. Growing awareness of the precursors to psychiatric disorder and to receiving support has led to increased early intervention and prevention strategies and a renewed focus on understanding pathways into care to reduce the ‘treatment gap’ and delays to care. The impact of improving earlier access to care and support could be considerable given that most psychiatric disorders emerge during childhood and adolescence, and it has been suggested that between a quarter and a half of adult cases might be prevented by effective earlier intervention among young people [1]. Thus, identifying ways to facilitate access to support early on could have important consequences for the life trajectories of young people with early signs and symptoms of mental illness.

Barriers to help-seeking occur throughout the development of mental illness [2, 3]. Self-recognition and awareness of one’s own mental health are a critical factor determining help-seeking at early stages of illness and also when experiencing severe symptoms and impairment over long periods of time [4, 5]. Individuals who do not recognise their difficulties as mental health problems do not direct their help-seeking towards mental health services [6]. Indeed, personal appraisal of one’s own mental health was shown to be a key predictor of perceived need for help among a community sample of adults meeting criteria for depressive disorder [5] and of subsequent help-seeking behaviour in a prospective study of persons with untreated mental illness [7]. Self-appraisal of having a mental illness could also be an important link which mediates the relationship between personal stigma and service use. It may be that individuals avoid the label of mental illness for themselves if they also have stigmatising views about people with mental illness [5, 8]. In addition, lack of self-recognition may also reflect individuals’ reluctance to accept a label of mental illness which could protect them from other negative self-perceptions associated with the label of mental illness and potential consequences of shame and reduced empowerment.

Given that most mental health problems emerge during childhood and adolescence, understanding the process of self-identification of mental illness is of particular interest amongst young people. Developing awareness and understanding of one’s mental health early on could also help young people to reflect on needed support, leading to a virtuous cycle for improving future engagement and mental health. Facilitating this cycle has the potential to influence the lives of many young people. For young people aged 10–24 years, mental illnesses also constitute the greatest cause of non-fatal burden of disease [9]. However, despite the high prevalence of mental health problems and their risk factors, most young people do not receive support for these difficulties: the prevalence of young people with mental illness who do not receive care appears comparatively larger for young people [10–12] and seems to be increasing [13–15].

An improved understanding of the process and factors underpinning people’s contact with mental health services could facilitate efforts to improve mental health care participation; however, little is known about how young people appraise their difficulties and self-identify as having a mental illness, particularly in relation with symptoms indicative of an increased risk of developing a psychiatric disorder. Assessing such self-identification has been a challenge as it is difficult to assess self-perceptions or experiences of stigma at these early stages of illness if individuals do not identify as having a mental health problem. Rather, studies have generally assessed a person’s knowledge, recognition or awareness of their symptoms as opposed to perceptions of these symptoms in relation with mental illness [16].

Recently, a measure was developed, in German, to assess adults’ appraisal of their present problem as mental illness [5]. In a German sample of adults with untreated mental disorders, the “self-identification as having a mental illness” scale (SELF-I) was shown to be associated with perceived need for help, intention to seek help, and help-seeking after 6 months [7]. Here, we present the psychometric properties of the English version of the SELF-I for use among young people in the community. We look at the relationship between self-perceptions in relation with having a mental illness in relation with psychiatric symptoms, service use, and intended stigmatising behaviour.

## Methods

### SELF-I instrument adaptation

We first translated the original German version of the questionnaire [5], in collaboration with the original scale developer (Schomerus). In collaboration with experts in the field of mental health stigma and measurement who reside in the UK (*n* = 5), we adapted the language to ensure that the items were relevant for the English context and maintained the initial conceptual idea. To ensure that the face validity and language were clear and relevant to young people in the UK, we consulted the Young People’s Advisory Panel from the Time to Change anti-stigma programme. The panel comprises young people aged 16–24 who have experience of mental health problems. The items were further adapted based on their feedback, and then, the subsequent version was checked for face validity among the original German team.

### Empirical testing of the adapted SELF-I

Following adaptation of the instrument, the revised SELF-I was piloted via phone interviews among a community sample of 423 young people. These data were collected as part of an ongoing prospective longitudinal investigation [17, 18]. The sample is enriched for risk of psychopathology as we over-sampled families from deprived, ethnically diverse inner-city areas, and included a higher proportion of young people with genetic and symptom-based risk factors for psychopathology [18]. This sample represents an ideal group for testing a measure of self-perceptions of mental illness as it comprises a high proportion of individuals with risk for psychopathology, including a wide range of symptoms, experiences and mental health service use (i.e., it includes individuals with and without mental health problems who have and have not used services and only a subgroup have received a psychiatric diagnosis). This is a significant strength over using a clinical sample who have all already received a formal diagnosis and/or label of mental illness. In addition, participants represent a broad continuum of mental health problems allowing us to relate varied subjective self-perceptions with the diverse symptom and impairment profiles captured in our sample. (Further detail on the sample can be found here [18].)

All young person participants provided written informed consent (written assent and parental consent for those who were under 16 years of age). The King’s College London and London School of Economics and Political Science Research Ethics Committees provided ethical approval for this study and it has, therefore, been performed in accordance with ethical standards laid down in the 1964 Declaration of Helsinki and its later amendments.

### Additional measures

We looked at SELF-I responses in relation with the following sociodemographic, clinical and service use characteristics: gender, age, neighbourhood deprivation (as measured by UK government index of multiple deprivation which is an indicator of social and economic deprivation across a 7 domains assessed at the postcode level [19]), psychiatric symptomatology (as measured by the Strengths and Difficulties Questionnaire [SDQ] [20]), psychotic-like experiences (as measured by the Psychotic-Like Experiences [PLEs] Questionnaire [21, 22] which is an adaptation and extension of items from the Diagnostic Interview Schedule for Children) and mental health service utilisation (as measured by the Services Assessment for Children and Adolescents [SACA] [23]) and intended stigmatising behaviour (as measured by the Reported and Intended Behaviour Scale [24]).

### Data analysis

#### Scoring of the SELF-I

SELF-I items were scored on an ordinal scale (1–5), where one represented ‘don’t agree at all’ and five represented ‘agree completely’. The total score for each participant was calculated by adding together the response values for all five items. Items 2, 4, and 5 were reverse coded when calculating total score or inter-item correlations, so that a higher score for all items was associated with a greater feeling of risk that one has or could develop a mental illness.

### Statistical analysis

Overall, each item’s psychometric performance was assessed by response frequencies and internal consistency reliability using Cronbach’s alpha [25]. Overall internal consistency was also measured in relation with each covariate. We invited 54 participants to complete the SELF-I twice, 2–3 weeks apart to assess test–retest reliability. For test–retest, a weighted kappa was performed for each item (assuming responses are ordinal). Lin’s statistic [26] was used to calculate the overall test–retest statistic for the entire SELF-I scale using the ‘concord’ command in Stata. We examined the association between sociodemographic (gender, age, and neighbourhood deprivation), clinical (SDQ, PLEs), and service use characteristics (within the healthcare setting or within the education setting), and intended stigmatising behaviour in relation with SELF-I responses, first using single linear regression models and next building multivariable linear regression models adding in groups of variables in blocks. Analyses were carried out using Stata version 14 and SAS version 9.4.

## Results

### Participant characteristics

More than half of participants were female (57%) and were of White ethnicity (59%). Age of participants ranged from 13 to 24 years, with a mean age of 18 (standard deviation 1.65). The distribution of neighbourhood deprivation was skewed, indicating that the majority of participants came from more deprived neighbourhoods. Fourteen percent of participants reported some type of mental health service use in the past year. Similar numbers of participants (just over 10%) reported some type of psychopathology or PLEs (see Table 1).

**Table 1:** Participant characteristics

	*n*	%
Gender		
Female	242	57.2
Male	181	42.8
Age		
13–17	147	34.8
18–20	251	59.3
21–24	25	5.9
Ethnicity		
White	245	58.5
Black	124	29.6
Asian	29	6.9
Other	21	5.0
Index of multiple deprivation decile		
1	20	4.7
2	88	20.8
3	56	13.2
4	54	12.8
5	49	11.6
6	53	12.5
7	31	7.3
8	39	9.2
9	21	5.0
10	12	2.8
SDQ classification		
Normal	376	89.3
Borderline	34	8.1
Abnormal	11	2.6
PLE		
No	363	89.0
Yes	45	(11.0)
Service use in past year		
Any service use	59	14.0
Health service use	51	12.1
Educational service use	14	3.3

*SDQ* Strengths and Difficulties Questionnaire; *PLE* psychotic-like experiences within the last year

### Feasibility and response patterns

Overall, participants tended to use the full range of response options (see Table 2), though responses tended to be slightly left skewed, indicating that the majority of respondents did not see themselves as having or at risk of developing a mental illness and this seems reasonable given the majority of participants did not present with psychiatric symptoms. Inter-item correlation coefficients were also moderate to substantial (range 0.44–0.73) suggesting that the items were addressing the same construct but in different ways that did not entirely overlap (see Appendix 1).

**Table 2:** SELF-I item responses, means, standard deviations, and test–retest reliability (*n* = 422)

	Do not agree at all *n* (%)	Do not agree *n* (%)	Undecided *n* (%)	Agree *n* (%)	Agree completely *n* (%)	Item mean (SD) *n* = 403	Kappa (*n* = 53)
1. Current issues I am facing could be the first signs of a mental illness	106 (25.1)	191 (45.4)	64 (15.2)	47 (11.1)	14 (3.3)	2.22 (1.05)	0.97
2. The thought of myself having a mental illness seems doubtful to me	21 (5.0)	81 (19.2)	78 (18.5)	202 (47.9)	40 (9.5)	3.38 (1.04)	0.93
3. I could be the type of person that is likely to have a mental illness	30 (7.1)	184 (43.6)	109 (25.8)	74 (17.5)	25 (5.9)	2.72 (1.03)	0.97
4. I see myself as a person that is mentally healthy and emotionally stable	7 (1.7)	40 (9.5)	62 (14.7)	232 (55.0)	81 (19.2)	3.81 (0.91)	0.90
5. I am mentally stable, I do not have a mental health problem	8 (1.9)	34 (8.1)	38 (9.0)	206 (48.8)	136 (32.2)	4.01 (0.95)	0.92
Total mean score*						11.74 (4.13)	0.95

*Items 2, 4 and 5 were reverse coded to calculate the total mean score

### Reliability

Overall test–retest reliability was high at 0.95 (see Table 2). We also examined item retest reliability using a weighted kappa, and this demonstrated high reliability for all items (range 0.90–0.97) over the retest period. The overall internal consistency as determined by the Cronbach’s alpha was 0.87, representing good internal consistency. In addition, when examining internal consistency according to sociodemographic, clinical and service use subgroups, Cronbach’s alpha remained above 0.75 (see Appendix 2). The inter-total correlations for each item, which ranged from 0.62 to 0.74, supported keeping all items in the scale as excluding any items from the overall scale would reduce the overall alpha and make the scale less reliable.

### Relationship between mental illness, service use, bullying, and self-perception as having a mental illness according to the SELF-I

Reporting greater psychiatric symptomatology via the SDQ and experience of PLEs was associated with a greater self-perception as having a mental illness (*p* < 0.05), providing evidence of convergent validity (see Table 3). We also found that mental health service use, in particular when it occurred in a healthcare setting, was associated with greater self-perceptions above and beyond symptomatology.

**Table 3:** Association between sociodemographic, clinical and stigma-related characteristics with self-identification as having a mental illness as measured by the SELF-I, linear regression models

	Model 1		Model 2	Model 3	Model 4	Model 5
	*β* (95% CI)	Adj. *R*-sq.	*β* (95% CI)	*β* (95% CI)	*β* (95% CI)	*β* (95% CI)
Sociodemographic characteristics						
Gender						
Male	− 0.27** (− 0.42, − 0.11)		− 0.27*** (− 0.43, − 0.11)	− 0.22** (− 0.37, − 0.08)	− 0.17* (− 0.31, − 0.03)	− 0.14* (− 0.28, − 0.002)
Female	Reference	0.03	Reference	Reference	Reference	Reference
Age group		0.02				
13–17	− 0.01 (− 0.22, 0.21)		0.02 (− 0.20, 0.23)	− 0.10 (− 0.29, 0.11)	− 0.07 (− 0.26, 0..11)	− 0.10 (− 0.24, 0.12)
18–20	0.1 (− 0.13, 0.27)		0.10 (− 0.13, 0.28)	0.01 (− 0.18, 0.20)	− 0.004 (− 0.18, 0.17)	− 0.01 (− 0.18, 0.16)
21–24	Reference		Reference	Reference	Reference	Reference
Ethnicity		0.001				
Black	− 0.05 (− 0.22, 0.13)		− 0.10 (− 0.25, 0.11)	− 0.10 (− 0.26, 0.08)	− 0.10 (− 0.26, 0.06)	− 0.06 (− 0.22, 0.10)
Asian	− 0.01 (− 0.32, 0.31)		− 0.02 (− 0.32, − 0.30)	− 0.10 (− 0.34, 0.23)	0.002 (− 0.26, 0.27)	0.06 (− 0.21, 0.32)
Other	0.04 (− 0.33, 0.40)		− 0.01 (− 0.32, 0.30)	− 0.10 (− 0.42, 0.26)	− 0.10 (− 0.40, 0.23)	− 0.05 (− 0.36, 0.26)
White	Reference		Reference	Reference	Reference	Reference
Index of multiple deprivation decile	− 0.02 (− 0.05, 0.01)	0.01	− 0.03 (− 0.06, 0.01)	− 0.02 (− 0.05, 0.01)	− 0.01 (− 0.04, 0.02)	− 0.02 (− 0.05, 0.01)
Mental health characteristics						
SDQ classification		0.10				
Abnormal	1.31*** (0.85, 1.77)			1.22*** (0.76, 1.67)	0.88*** (0.46, 1.30)	0.82*** (0.40, 1.23)
Borderline	0.58*** (0.31, 0.85)			0.47*** (0.20, 0.74)	0.32* (0.07, 0.57)	0.36** (0.12, 0.61)
Normal	Reference		Reference	Reference	Reference	Reference
PLE total score	0.65*** (0.40, 0.89)	0.06		0.48*** (0.24, 0.73)	0.36** (0.14, 0.58)	0.37* (0.14, 0.59)
Service use in past year						
Health service use	1.18*** (0.97, 1.39)	0.23			0.93*** (0.72, 1.15)	0.92*** (0.71, 1.13)
Education service use	0.83*** (0.38, 1.27)	0.03			0.33 (− 0.09, 0.76)	0.36 (− 0.06, 0.79)
Stigma variables						
RIBS total score	0.23*** (0.10, 0.37)	0.04				***0.18 (0.07, 0.28)
Adj. *R*-sq.			0.04	0.17	0.32	0.34

*SDQ* Strengths and Difficulties Questionnaire, *PLE* psychotic-like experiences within the last year, *RIBS* reported and Intended Behaviour Scale

*p < 0.05, **p < 0.01, ***p < 0.001

## Discussion

The aim of this study was to present the psychometric properties of the English version of the SELF-I for use among young people in the community and to examine how these reported self-perceptions of mental illness are related to sociodemographic, clinical, and stigma-related characteristics. The SELF-I demonstrated robust psychometric properties including high test–retest reliability and good internal consistency. The SELF-I represents a brief instrument to assess an individual’s subjective perception of their own identity in relation with mental illness, a key intermediary between stigma and help-seeking, which could easily be added to existing population surveys with minimal participant burden.

The robustness of the psychometric properties of the SELF-I in a fairly heterogeneous community sample provides confidence that the measure could be applied robustly to a variety of samples. The overall alpha of 0.87 is well above the minimum threshold of 0.7 [27, 28], and three, if there was a decrease in consistency among a more heterogeneous sample, this would still not pose a problem. Moreover, subgroup analyses of internal consistency by sociodemographic, clinical, stigma, and service use-related subgroups were all over 0.7. The lowest value was reported among those who reported no mental health symptomatology on the SDQ, suggesting that there is slightly greater inter-item response variation in this group, possibly because these individuals might have been less likely to previously consider or think about their own mental health [8]. Those with less personal experience are less likely to consider issues of mental illness [29].

The assessment of self-perception of having a mental illness among young people is important in that it can aid our understanding of how individuals, who may not identify as a service user or be engaged with clinical services, perceive their own mental state and potential risk for developing a mental illness. Given that self-perceptions have shown to represent a key mediating factor between stigma and help-seeking [7, 30, 31], it could also help us to understand this pathway between stigma and help-seeking, and associated factors or target groups who experience this as a more significant barrier. As we expected, our data showed that mental health symptoms were associated with greater self-perceptions of having a mental illness as measured by the SELF-I; however, other factors were also associated with greater self-perceptions including: being female, using mental health services in the past year and having less intended stigmatising behaviour in relation with people with mental illness and mental health service use, independent from mental health problems.

It is likely that those with more intended stigmatising behaviour would be more likely to avoid the label of mental illness, while the experience of using mental health services could reinforce a label or identity of having a mental illness either through conferring a diagnosis or because of the implications around crossing a threshold of needing treatment [30]. Indeed, labelling oneself as having a mental illness can be a double edged sword. Although recognition of having a mental illness is a key step to accessing support and/or treatment [32], self-labelling oneself as having a mental illness can also introduce additional distress and enable self-stigma. Application of the label of mental illness to oneself could activate negative perceptions of people with mental illness and lead to reduced self-esteem and self-efficacy and increased shame. Although this relationship is established in the literature [33, 34], we also know that this process is context dependent and is mitigated by reducing public stigma [35–37]. Thus, a key step in reducing the effects of labelling is also reducing public stigma, so that the negative views are not internalised. Other research notes that stigma resistance can also be an effective antidote to withstanding the negative effects of labelling [38]. Interventions which aim to increase help-seeking through increased self-recognition might also consider incorporating features which increase stigma resistance and reduce public stigma [39]. Thus, we would not recommend a programme solely to enhance self-identification of having a mental illness based on our results. We think that the SELF-I, however, could represent an important assessment tool to better understand the process of self-recognition and help-seeking.

Our results are similar to those elicited in a sample of German adults with untreated mental illness. In this German group, personal stigmatising attitudes were associated with lower self-identification, while higher mental health literacy and having been previously treated for mental health problems seemed to facilitate greater self-perception as having a mental illness. Higher self-identification at baseline also predicted help-seeking from a mental health professional during a 6-months follow-up period [7].

There are some limitations to this study which should be considered. First, our study sample was not representative of Greater London, but rather included a convenience sample of students attending schools which overrepresented deprived, ethnically diverse neighbourhoods. As we assessed the SELF-I among participants in the third wave of follow-up, we found that participants with higher psychopathology and those who reported their ethnicity as other than white were underrepresented in wave 3 in comparison with the original community sample. Nevertheless, we think that the validity of the identified relationships between SELF-I items themselves and in relation with other measured variables remain valid. As previously discussed, a more heterogeneous sample could dilute some of the psychometric properties, yet given the statistics were well above recommended guidelines, it would seem that they would remain substantial even among more varied samples. Given all participants were residents of the Greater London area, however, we do not know how our findings would translate to other contexts. Additional limitations are that although mental health service use and psychiatric symptomatology were assessed via validated instruments (i.e., the SACA and SDQ, respectively), these are self-report measures. Nevertheless, the data suggest that the relationships go in the expected direction in that greater mental health symptomatology was related with a higher likelihood of service use, and both these measures were related to greater self-perceptions of having a mental illness.

Despite these limitations, the SELF-I represents a unique instrument which allows for investigating subjective perceptions of one’s own mental health status among community samples. In particular, the SELF-I could be a useful tool for understanding self-perceptions among high-risk populations or non-help-seeking populations which do not necessarily identify as having a mental illness. Additional research should explore stability versus flexibility of self-perceptions over time, in particular alongside the development and/or recovery from mental health problems and how it relates to anti-stigma interventions.

## Appendix 1

**Table:**

Inter-item correlation coefficients (*n* = 423)		1	2	3	4	5
1	Item 1	1				
2	Item 2—r	0.50***	1			
3	Item 3	0.58***	0.60***	1		
4	Item 4—r	0.62***	0.44***	0.54***	1	
5	Item 5—r	0.63***	0.50***	0.58***	0.73***	1

*r* items were coded inverse

****p* < 0.001

## Appendix 2

**Table:**

	Cronbach’s alpha
Total sample	0.87
Gender	
Female	0.87
Male	0.87
Ethnicity	
White	0.85
Black African or African-Caribbean	0.91
South Asian or Oriental	0.79
Other	0.82
Index of multiple deprivation decile	
1–2	0.87
3–4	0.85
5–6	0.85
7–8	0.92
9–10	0.82
SDQ classification	
Normal	0.77
Borderline	0.86
Abnormal	0.90
PLE	
No	0.87
Yes	0.92
Service use in past year	
Health service use = yes	0.89
Health service use = no	0.82
Educational service use = yes	0.78
Educational service use = no	0.87

*SDQ* Strengths and Difficulties Questionnaire, *PLE* psychotic-like experiences within the last year
